# A Dissertation on the Diseases of the Dental Pulp and Their Treatment

**Published:** 1854-07

**Authors:** Chapin A. Harris


### ARTICLE II.

*A Dissertation on the Diseases of the Dental Pulp and their Treatment*, prepared for, and read before, THE ASSOCIATED ALUMNI OF AMERICAN DENTAL COLLEGES, at the BALTIMORE COLLEGE, March 18, 1854. By CHAPIN A. HARRIS, M. D., D. D. S.

[Continued from page 404.]

*Inflammation.*—The pulp of a tooth, when healthy, has a grayish-white appearance, and its capillaries are invisible to the naked eye, but when it becomes the seat of *acute* or *active* inflammation, they may be distinctly seen—the organ having assumed a bright red color. Inflammation having established itself, soon extends to every part of the pulp, and even to the alveolo-dental periosteum. When permitted to run its course uninterruptedly, it usually terminates in suppuration in from three to eight or ten days.

The unyielding nature of the walls of the cavity in which it is, on all sides, enclosed, renders expansion of the pulp impossible, and as its capillaries become distended with blood, they press on the nervous filaments which are every where distributed upon it, causing at first constant *gnawing*, but afterwards, as the distension of the vessels increases, severe, and sometimes almost insupportable, *deep-seated*, *throbbing* pain.

Inflammation may attack the pulps of sound teeth as well as those that are affected with caries, but it occurs more frequently in the latter than in the former, and it is oftener met with before than after the pulp has become actually exposed. The severity of it, however, is determined by the condition of the tooth, the state of the general health and the causes concerned in its production. The pulp when in an irritable condition is more liable to become the seat of acute inflammation that when in a perfectly healthy state, and the occurrence of suppuration is soon followed by alveolar abscess, unless an opening is made immediately through the crown, neck or root of the tooth for the escape of the matter.

The effusion of lymph which takes place during the inflammatory stage, and which, under other circumstances, and when the inflammation is less severe, is made to play an important part in the reparation of the injury, compresses the pulp into still narrower limits as it accumulates in quantity, and thus, becomes an additional source of irritation, adding fuel to the flame already lighted up.

Inflammation of the pulp may be caused by a blow on the tooth; by impressions of heat and cold conveyed to it through the conducting medium either of the enamel and dentine, or a metallic filling, or by the pressure of a filling, or the direct contact of external irritating agents, as disorganized portions of the tooth, particles of alimentary substances, acrid humors, &c., But as I have stated in another place, *inflammation* of the dental pulp "is not always a necessary consequence of impressions" of heat and cold; "pain may be produced by them when it does not exist, but in this case, it usually subsides soon after the removal of the irritant. The pulp of a tooth may be exposed for months, and subjected several times a day to the actual contact of foreign bodies, without becoming the seat of acute inflammation. The irritation, and increased vascular action thus occasioned, are, no doubt, removed by the effusion of lymph to which they give rise, and the pulp, after it has become exposed, having room to expand as its vessels become distended, does not suffer irritation from the pressure to which it would otherwise be subjected.

When suppuration takes place, the pain very nearly ceases, but the tooth for a time remains sore to the touch, and its appearance is changed. It has no longer the peculiar animated translucency of a living tooth, but has assumed an opaque, muddy or brownish aspect. With the disorganization of the pulp the entire crown and inner walls of the root lose their vitality; still, if the alveolo-dental periosteum has not become seriously involved in the disease, the vascular and nervous supply furnished the exterior of the root, is often sufficient to prevent the tooth from exerting a manifestly obnoxious influence upon the surrounding and more highly vitalized parts. The cementum being more analogous in structure to true osseous tissue than dentine, now plays an important part in the animal economy. It being more liberally supplied with the nutritive juices and vitality, and not being sensibly affected by the death of the other parts of the organ, it keeps up the living relationships of the tooth with the alveolo-dental periosteum, at least sufficiently to prevent it from acting perceptibly as a morbid irritant.

Inflammation of the pulp of a tooth, besides the local pain with which it is attended, often gives rise to a train of constitutional morbid phenomena, usually of a mild, but sometimes of an aggravated and even threatening character. Among these are *head-ache*, *constipation of the bowels*, *furred tongue*, *dryness of the skin*, *quick*, *full* and *hard pulse*, *ear-ache*, *ophthalmia*, *disease of the maxillary sinus*, &c. The following cases, taken from many of a similar character, which have fallen under my immediate observation, will serve to convey some idea of the effects liable to result from this cause.

Mr. H——, a resident of Baltimore, of a sanguino-nervous temperament, about thirty-five years of age, had a left bicuspid of the upper jaw filled in May, 1850. The cavity in the tooth, as I afterwards ascertained from an examination, extended nearly to the lining membrane. The only immediate inconvenience experienced from the operation, as I was informed, was a slight momentary pain whenever hot or cold fluids were taken into the mouth. The tooth remained in this state for about two months, but at the expiration of this time, it began to ache. The pain, however, being slight, was attributed to a cold contracted a short time before, but in a few days it increased and soon became so severe as to be almost insupportable, depriving him of sleep at night. The pulp had now become the seat of active inflammation, which rapidly extended to the socket and gum, and in a short time to the alveoli of three or four of the neighboring teeth and to the maxillary sinus.

Congestion of the brain, with all its attendant phenomena, such as fever, full, hard and quick pulse, intolerance of light, and slight delirium supervened.

The most active treatment was promptly instituted and energetically pursued, consisting of copious blood-letting from the arm, saline purgatives in large doses, blisters to the back of the neck, leeches to the gums, and the extraction of the tooth which had given rise to the disturbance; but, notwithstanding, the local inflammation terminated in suppuration and necrosis of the sockets of five teeth and the floor of the maxillary sinus. The constitutional symptoms, in the meantime, disappeared, and at the expiration of about eight weeks, the dead bone had so far separated from the living that I was enabled, without difficulty, to remove it.

Now, the whole train of morbid phenomena in this case, originated in irritation produced by impressions of heat and cold, conveyed to the pulp through the medium of a filling in a tooth, and a thin layer of dentine. But in a person of less nervous excitability, these impressions would not have given rise to any permanent local disturbance, and they would not have been felt at all, if a non-conducting substance had been placed in the bottom of the cavity previously to the introduction of the filling. The prompt removal of the filling would have prevented the painful consequences that supervened, but when I first saw the patient, suppuration had commenced, and hence the removal of the tooth failed to afford relief.

In December, 1849, Mr. M., about thirty years of age, of sanguino-bilious temperament, sedentary habits, contracted a severe cold. The next day the left cuspid tooth in the upper jaw became the seat of intense throbbing pain. This, in common with all his other teeth, had sustained considerable loss of substance from mechanical abrasion, owing to the manner in which the teeth of the upper and lower jaws came together, but it had not suffered injury from any other cause. The inflammation which had seized upon the pulp soon extended to the alveolo-dental periosteum and gums. High fever, constipation of the bowels, and inflammation of the conjunctival membrane of the eye of the affected side rapidly supervened. His sufferings, for several days, were intense.

The patient had been troubled occasionally, for several years previous to this attack, with dyspepsia, and at such times he experienced pain in several of his teeth whenever he took hot or cold fluids into his mouth, or inhaled cold air, but as it subsided immediately, it excited no apprehension, especially as the teeth in which these impressions were felt were free from caries. I saw the patient for the first time the evening of the third day after the attack. Suppuration of the pulp had now taken place; the tooth was slightly pushed from its socket, and the gum around it was swollen, indicating that alveolar abscess was in progress of formation.

The most active means had been used to arrest the inflammation and put a stop to the threatening constitutional symptoms, but as they were not resorted to until suppuration of the pulp was about taking place, they afforded but little relief. To give egress to the confined pus seemed now to constitute the first and most important curative indication, and for this purpose a hole was drilled through the worn end of the tooth to the pulp cavity. The escape of matter that followed the withdrawal of the instrument gave immediate relief. The inflammation of the surrounding parts, and of the eye, soon disappeared, and in a week the gum had assumed a perfectly healthy appearance.

In this case, nature, from some cause which cannot be easily explained, had failed to make that provision against the exposure of the pulp which she usually does under similar circumstances, consisting, as has already been intimated, in the gradual conversion of this organ, as the abrasion approaches the central cavity, into *osteodentine.*

The subject of the third case was a young lady of a sanguinous temperament, about nineteen years of age. In the summer of 1852, a small filling was put in the left approximal surface of a lateral incisor, after having been first separated from the adjoining central. Slight, transient pain was experienced for a few days after the operation, whenever she took hot or cold fluids into her mouth, but this only occasioned momentary inconvenience, the unpleasant sensation ceasing almost immediately. In the fall of 1853, after a slight attack of congestive fever, during convalescence and after her recovery, the pulp of the tooth was so exceedingly irritable that the inhalation even of cold air caused severe pain. In a few weeks inflammation appeared, and although leeches were promptly applied to the gum, and saline purgatives prescribed, its intensity was not abated in the least; it soon extended to the alveolus. The local disturbance, in this instance, not only caused the disorganization of the pulp, but it was also attended by an attack of inflammatory fever.

Mr. Billisurio, dentist, of Sydney, Australia, relates, in the American Journal of Dental Science, the case of a man twenty-five years of age, who, after a "severe wetting," while on his passage from England to that colony, when off the cape, had the pulps of his lower incisors, cuspids and bicuspids attacked by inflammation, which terminated in each tooth in suppuration and alveolar abscess.

The amount of constitutional disturbance arising from inflammation of the pulp of a tooth, depends on the state of the general health, and the nervous irritability of the system at the time. In the majority of cases it occasions but little inconvenience, and disappears as soon as the inflammation ceases, but sometimes it assumes a very alarming character. A case of fatal tetanus, produced by inflammation of the pulp of a lower molar, occurred a few years ago in Baltimore. The subject was a young lady about eighteen years of age. The system, at the time, from great bodily fatigue and mental excitement, was in an exceedingly irritable condition, but in other respects, though constitutionally rather delicate, she was in the enjoyment of good health.

There is not an organ or tissue of the body in which acute inflammation is more intractable in its nature, and rapid in its progress than in the pulp of a tooth; and when we take into consideration its situation, and physical and vital peculiarities, it is not to be wondered, that it should, in so large a majority of the cases, terminate in the disorganization of the part.

Still, it may sometimes be arrested, and the remedial indications here, though they cannot be as readily and fully carried out, are the same as for inflammation in any other part of the body. The first and most important one consists in the removal of all local and exciting causes. If it be the result of irritation produced by the pressure of a filling, the plug should be immediately removed, leeches applied to the gum of the affected tooth, and, if the patient be of a full habit, blood may be taken from the arm, and a brisk saline purgative prescribed. The removal of the filling, however, when the inflammation has previously made much progress, will not prevent suppuration, but it may prevent it from extending to every part of the pulp. When an external opening is made for the escape of the matter the moment suppuration takes place, the remaining portion of the pulp will be relieved from pressure, the cause of the irritation, and then the inflammatory action may cease. But if the matter remains in the central cavity of the tooth, the part of the pulp which has not suppurated will still be subjected to pressure, and the inflammation and suppuration will go on until the entire organ perishes. Nor will the disorganizing process stop here. The alveolo-dental membrane at the extremity of the root, will soon become implicated, and in a short time alveolar abscess will form, thus terminating the acute stage of the disease.

There may be no indications of irritation or inflammation for several weeks, or even months, after a tooth has been filled, but at the expiration of this time, the pulp, from increased irritability, caused, perhaps, by some change in the state of the patient's general health, may be attacked by inflammation. Although this very seldom happens, it does, nevertheless, sometimes occur, and when there is reason to apprehend that it is about to take place, and it may be suspected if pain is felt in the tooth when anything hot or cold is taken into the mouth, or if it becomes the seat of gnawing or gradually increasing pain, the filling should be removed. If the pain now ceases, a thick layer of gutta percha, or "Hill's stopping" may be placed in the bottom of the cavity, and the filling replaced, using the precaution as before directed, to introduce the gold in such a way as to prevent the liability of depressing the floor of the cavity. But if the pain and inflammation should continue unabated, it may be necessary to extract the tooth, or expose the pulp and destroy its vitality by applying to it some powerful escharotic, as the arsenious, which, acting more promptly and with more certainty than any other, seems best adapted to the purpose. When this is done, it is usually with the view of securing the retention and preservation of the tooth by filling the pulp cavity and root, an operation now very frequently performed by many dentists.

The abstraction of blood directly from the pulp, one would suppose, would be better calculated to arrest inflammation of this organ than almost any other treatment, but I do not think this has been resorted to for this purpose sufficiently often to determine the amount of therapeutic agency it is capable of exerting. At any rate, it seems reasonable to suppose that if, by this means, the congestion of the capillaries could be removed, the tumefied pulp would be reduced to its natural size, and be relieved from the pressure to which, as a consequence of its distended condition, it was subjected. To obtain the largest amount of benefit capable of being derived from the operation, the puncture should be made in that portion where one of the principal arteries would be most likely to be punctured, and this, it seems to me, would be just where the canal of the root enters the chamber of the crown of the tooth. But in making the puncture here, the pulp being very small at this point, there is danger of cutting it off, and as reunion would scarcely be likely to take place, the portion in the central cavity would necessarily perish. I have made the operation *(odontry'py)* three times, and in two of the cases it at first appeared to have been successful, it having been followed by immediate cessation of pain, but this, as I afterwards ascertained, was either owing to the complete division, or the division of so large a portion of the pulp, that the part in the crown soon died, the drill having entered the canal in the root a little above the chamber of the the tooth. In the other case, the inflammation having never reached its height, the operation increased the severity of the pain, and an hour or two after its performance, at the earnest solicitation of my patient, I extracted the tooth.

If the pulp were exposed, there would be a better opportunity of relieving the congested condition of its capillaries by the abstraction of blood, but the difficulty of obtaining free access to the organ by drilling a hole through the intervening dentine is so great, the tooth, when suffering from inflammation, being usually so sore to the touch, that the slightest pressure is productive of great pain, hence, the operation will seldom if ever prove successful. Unless, therefore, the retention of the tooth is a matter of more than ordinary importance, it is better to remove it at once. But if it is an incisor or cuspidatus, the pulp should either be immediately extirpated or arsenious acid applied for the destruction of its vitality; or, if suppuration has previously taken place, an opening should be made into the chamber of the tooth as before directed, for the escape of the matter. Should it be found, after this has escaped, that disorganization has not extended to every part of the pulp, the remaining portion may be destroyed in the manner as above described. This done, the pulp cavity and root, as soon as the inflammation of the socket has completely subsided, may be filled.

It will be seen from the foregoing remarks, it is only at its very inception, that there is any chance of combating successfully acute inflammation of the pulp of the tooth, and even then, so rapid is the progress of the disease, it may baffle the best directed and most energetic treatment that can be adopted. It may be that when attention shall have become more generally directed to the subject, that some more successful method of treatment may be discovered, but that a complete mastery over the disease will ever be obtained, is not to be expected.

But inflammation of the dental pulp is not always acute; it sometimes assumes a chronic and local form. This often occurs where the chamber of the tooth has become gradually exposed by caries of the dentine, and when it happens, the action of the fluids of the mouth, and other foreign substances which obtain access to the cavity, as well as the decomposed portions of the tooth substance, cause an increase of vascular action in the exposed part, followed, very often, by a slight discharge, but the morbid action thus induced is, comparatively seldom, accompanied by pain. The pulp may remain thus partially exposed for months, and even years, without causing any other inconvenience than a momentary twinge of pain when some hard substance is accidentally introduced into the cavity of the tooth, which subsides immediately after its removal. Sooner or later, however, the pain thus excited will become more permanent, continuing each time it is produced, from five or ten minutes to one or more hours after the cause of the irritation has been removed. If a tooth be filled under such circumstances, the pressure of the fluid upon the pulp, which is poured out from its exposed surface beneath the plug, will give rise to a more general and active form of inflammatory action.

The liability of the tooth to ache increases as the pulp becomes more and more exposed by the gradual decomposition of the dentine, and the inflammation may ultimately assume a more active form, or the pulp may become the seat of fungous growth, or be absorbed or destroyed by ulceration, or gangrene and mortification. Cases sometimes occur in which the disease is attended with severe darting pains, occurring, very often, several times in the space of two or three minutes, succeeded by intervals of perfect ease of as many hours. At other times it is attended by dull aching pain, which is aggravated by taking sweet or acid substances into the mouth. In cases of this sort, the application of heating or stimulating substances to the exposed surface of the pulp, will usually procure relief. Permanent exemption from pain, however, is rarely obtained, and sooner or later, it becomes necessary either to destroy the pulp or extract the tooth.

The body of the pulp, when the organ becomes exposed from a decayed opening in the grinding surface of a molar, is sometimes absorbed, while the prolongations in the roots often remain unchanged for two, three or more years. But when it becomes exposed from an opening in, and confined to, any of the other surfaces of the crown, it is rarely thus removed.

Chronic inflammation of an exposed surface of the pulp, when long continued, sometimes gives rise to *ulceration*—a disorganizing process which often causes the destruction of a large portion of the part occupying the central chamber of the crown of the tooth, making in it numerous little excavations. The ulcerated surface usually presents a yellowish appearance, and when the disorganizing process is arrested before it has effected the destruction of a very large portion of the pulp, it usually becomes covered with healthy granulations.

When the inflammation occurs in cachectic individuals it often assumes an acute form, and sometimes terminates in gangrene and mortification. The loss of vitality may be confined to the body of the pulp, or it may extend to every part of the organ. In the former case the pain continues, but in the latter it ceases as soon as mortification takes place. When this happens, the entire pulp, which has now a dark brown or black color, may be removed. But this is not a very common termination.

The symptoms of chronic as well as acute inflammation are always modified by the state of the general health, habit of body, and the temperament of the individual. The pain attending the former, however, is periodical, occurring at irregular and uncertain intervals, and constitutes that variety of tooth-ache so often relieved by local applications, whereas, in the latter, it is constant.

In chronic inflammation, the pulp is either actually exposed or only covered by decomposed or partially decomposed dentine, and the diseased surface rarely embraces a larger circumference than that described by the bottom of the decayed cavity. The inflammation, therefore, is local as well as chronic, but nevertheless, it is often of so persistent a character, as to render its removal exceedingly difficult. The dentist, however, is not so much restricted in the application of remedies as in the treatment of acute inflammation, and to the action of which it yields more readily. But notwithstanding all this, he will necessarily encounter difficulties in his efforts to subdue it. A greater length of time is sometimes required than the patient is willing to give, and the opening through the crown to the central cavity is, not unfrequently, too small, previously to the removal of the partially decomposed dentine, to admit of the direct application of the necessary remedial agent to the inflamed surface of the pulp. Again, it often happens, that the situation of the tooth and cavity are such as to prevent him from obtaining a complete view of the diseased part, and it is important that he should do this to enable him to determine whether the inflamed surface is ulcerated, or pours out a serous fluid, or whether the morbid condition consists merely of irritation, produced by the presence of acid matter, or of partially or wholly decomposed dentine. Unless his diagnosis is correct, his prescription will be as likely to do harm as good. But, having ascertained the exact character of the disease, he may often be able to institute treatment that will result in the restoration of the pulp and the preservation of the tooth.

It is important, too, that he should understand the part which nature plays in the curative process, for cure here, as in the case of the cure of disease in other parts of the body, is effected by that internal force, which, as Chomel says, "presides over all the phenomena of life, contends unremittingly with physical and chemical laws, receives the impression of deleterious agents, reacts against them and effects the resolution of disease." This vital force is sometimes efficiently exercised for the cure of disease in the pulp of a tooth, but more frequently for its prevention, as is shown by the gradual ossification of the organ in those cases where it would otherwise become exposed by mechanical or spontaneous abrasion of the solid structures which enclose it, and occasionally by the formation of new dentine upon its surface at a point towards which caries is advancing. Nature, no doubt, would always provide in this way against the exposure of the pulp, if the occurrence was always preceded for a sufficient length of time to enable her to do so, by sufficient irritation or increase of vascular action in it to call her energies into operation. But the formation of new dentine, which constitutes the protective wall of defence, is a tardy process, and as a general rule, proceeds more slowly than the caries in the tooth, which causes the exposure of the pulp. Besides, it often happens that its approach is not announced by the slightest irritation, a condition necessary to the new formation, until it reaches the central cavity. At other times, the approach of the disease gives rise to too much irritation, a condition equally unfavorable to ossification of the pulp. Thus no protective covering being formed, it soon becomes exposed, when it is subjected to the action of such irritating agents as may chance to be brought in contact with it. Hence, its liability to become the seat of chronic inflammation as well as other forms of diseased action.

If the disease be attended with pain, the removal of this should first claim attention, and this should be effected with as little delay as possible, otherwise the morbid action may extend to every part of the pulp and peridental membrane, and assume a more active and unmanageable form. If the pain is the result of irritation produced by the direct action of mechanical or chemical agents, the cavity in the tooth should at once be carefully freed from all extraneous substances and the decomposed portions of dentine. This done, a dossil of raw cotton or lint, saturated with spirits of camphor, laudanum, sulphuric ether, chloroform, creosote, or some one of the essential oils may be applied. Immediate relief is sometimes obtained by an application of this sort. Counter irritants have sometimes been used with advantage. The pain has often been removed by exciting increased secretion of saliva, but when a sialagogue is used, the cavity in the tooth should be filled with raw cotton or lint to prevent the agent from being brought in contact with the exposed surface of the pulp. But a remedy which will relieve the pain in one case often aggravates it in another.

When the irritation is produced by acidulated buccal fluids, the application of carbonate of soda or some other alkali, will often give immediate temporary relief, but as the condition of the secretions of the mouth, especially of the salivary, is usually owing to gastric derangement, the correction of this constitutes the first and most important remedial indication. When any application is made to the pulp for the purpose of removing irritation and pain, their full effect will not be obtained unless the fluids of the mouth are excluded from the cavity of the tooth, and this may be done by closing the orifice with softened wax or mastic, using the precaution not to force it in so far as to press the application, previously introduced, upon the nerve.

Chloroform, from its powerful anæsthetic properties, is now regarded as one of the most efficient antiodontalgic agents that has ever been employed, especially in those cases where the pulp of the tooth is actually exposed. I have used it, after having dissolved gutta purcha in it until it was of the consistence of molasses, with the most satisfactory results, and in two cases, after the pulp had become the seat of chronic inflammation, with complete success. One of which I will describe.

Mrs. W——, wife of a medical gentleman residing near Baltimore, of a sanguino-bilious temperament, and about twenty-eight years of age, applied to me in the summer of 1851, for my professional aid. The second molar on the right side in the lower jaw had a large cavity in the grinding surface, which, at one point, had penetrated to the pulp. The tooth having frequently ached, I advised her to have it removed, but she could not be persuaded to submit to the operation. After removing the foreign matter and decayed dentine, the exposed surface of the pulp presented a reddish appearance, the capillaries of this part having become injected with red blood. The tooth was aching at the time, and under these circumstances, the cavity was filled with raw cotton, saturated with a thick solution of gutta percha in chloroform. The pain ceased immediately, and a few minutes after she left my office, promising to return and have the tooth extracted if it should again become painful. Two months elapsed before I saw her, and up to this time her tooth had given her no further trouble, the cotton having remained in it during the whole period of her absence, and it was with some difficulty that I succeeded in removing it, as it was firmly imbedded in the gutta percha, which had remained after the chloroform had evaporated. I now dried the cavity carefully and filled it with Hill's stopping, leaving a small space between the filling and the exposed pulp. At the expiration of about eight months, agreeably to my request, she called on me again, when I replaced the temporary filling with a permanent one of gold, having previously, however, placed a thin layer of gutta percha on the bottom of the cavity. The tooth has subsequently remained free from pain.

Until within the last three or four years, I did not believe it possible to preserve the vitality of a tooth by filling after the pulp had become the seat of chronic inflammation, but am now convinced that it can be done in very many cases, but to effect which several weeks of preparatory treatment are often required. I have succeeded in fully one-half of the cases which I have treated since the commencement of 1852, and it is probable, that when the pathology and remedial indications of the diseases of the dental pulp shall be better understood, greater relative success may be had in the treatment of the disease in question. The practicability of restoring teeth to health and usefulness after the pulp has become the seat of pain and actual disease, has hitherto been regarded by all, as beyond the reach of the dentist's skill.

In a conversation with Dr. W. W. Codman, of Boston, in 1850, this gentleman informed me, that he had succeeded in inducing ossification of the dental pulp by removing the decomposed dentine and keeping the cavity in the tooth filled with raw cotton. The time required to effect this, varying from eight to fifteen months, and during this period he directs that the cotton be renewed, at least, once a day. By this simple treatment, Dr. C. assured me that he had succeeded in numerous instances in exciting ossific inflammation, and a bony covering having formed over the pulp, he filled the cavity in the tooth without fear of subsequent trouble. Whether this treatment was adopted in cases where chronic inflammation of the pulp existed, or only where this organ was in a healthy condition, I am not able to say, as I do not recollect that the subject was alluded to at the time of our conversation. Dr. W. H. Dwinelle, of Cazenovia, N. Y., recommends the use of tannate of lead and spirits of camphor, which seems to have been more successful in his hands than most other remedial agents employed for irritation or inflammation of the dental pulp. He recommends the use of friction with a view of exciting ossific inflammation, and this may sometimes be attended with a very good effect, especially, if the morbid action is dependent in part upon want of sufficient vital energy in the pulp to induce a deposit of bony matter on the exposed surface.

[To be Continued.]

